# Remifentanil Protects against Lipopolysaccharide-Induced Inflammation through PARP-1/NF-*κ*B Signaling Pathway

**DOI:** 10.1155/2019/3013716

**Published:** 2019-12-31

**Authors:** Jian-ning Zhang, Yang Ma, Xi-yan Wei, Ke-yin Liu, Hao Wang, Hui Han, Yi Cui, Ming-xiang Zhang, Wei-dong Qin

**Affiliations:** ^1^Department of Critical Care Medicine, Qilu Hospital of Shandong University, Jinan, Shandong, China; ^2^State Key Laboratory of Biobased Material and Green Papermaking, Key Laboratory of Pulp & Paper Science and Technology of Shandong Province/Ministry of Education, Qilu University of Technology, Shandong Academy of Sciences, Jinan, China; ^3^The Key Laboratory of Cardiovascular Remodeling and Function Research, Chinese Ministry of Education and Chinese Ministry of Public Health, Qilu Hospital of Shandong University, Jinan, Shandong, China

## Abstract

Sepsis is a leading cause of death in patients with severe infection worldwide. Remifentanil is an ultra-short-acting, potent opioid analgesic. In the study, we aimed to investigate the role and underlying mechanism of remifentanil in lipopolysaccharide- (LPS-) induced inflammation in human aortic endothelial cells (HAECs). HAECs were pretreated with phosphate-buffered saline (PBS) or remifentanil (2.5 *μ*M) for 30 min, then stimulated by LPS (10 *μ*g/ml) for another 24 h. Poly(ADP-ribose) polymerase 1 (PARP-1) was inhibited by small interfering RNA (siRNA). Superoxide anion production and DNA damage were analyzed by dihydroethidium (DHE) staining and comet assay. The inducible nitric oxide synthase (iNOS), intercellular adhesion molecule 1 (ICAM-1), PARP-1, poly(ADP-ribose) (PAR), and nuclear factor-kappa B p65 (NF-*κ*B p65) expressions were analyzed by RT-PCR or western blotting analysis. NF-*κ*B p65 nuclear translocation was assessed by immunofluorescence. Compared with the control group, pretreatment with remifentanil significantly reduced superoxide anion production and DNA damage, with downregulation of iNOS, ICAM-1, and PARP-1 expressions as well as PAR expression. Moreover, pretreatment with PARP-1 siRNA or remifentanil inhibited LPS-induced NF-*κ*B p65 expression and nuclear translocation. Remifentanil reduced LPS-induced inflammatory response through PARP-1/NF-*κ*B signaling pathway. Remifentanil might be an optimal choice of analgesia in septic patients.

## 1. Introduction

Sepsis occurs and results in life-threatening tissue damage and organ dysfunction when a dysregulated host response to an infection [1]. It is a serious condition with a mortality of 15–20% and a short- and long-term morbidity [2, 3]. Sepsis is characterized by not only increased inflammation response but also immune suppression [4]. The effects of inappropriate response to infection lead to cellular dysfunction and organ failure. Lipopolysaccharide (LPS), the major constituent of the outer cell wall of Gram-negative bacteria, is known to induce the production of various inflammatory cytokines, which are recognized as principal components in the cause of sepsis [5]. Although many studies have been designed to investigate the mechanisms and therapeutic approaches of sepsis or septic shock, its etiology remains unclear and the prognosis is still poor.

Poly(ADP-ribose) polymerase-1 (PARP-1) is a nuclear enzyme that is activated by DNA damage [6]. It is involved in the regulation of key transcription factors, such as nuclear factor-kappa B (NF-*κ*B), to regulate gene expressions, including intercellular adhesion molecule 1 (ICAM-1) and inducible nitric oxide synthase (iNOS), and exerts a profound inflammatory response [7, 8]. A large number of studies have well addressed the involvement of PARP-1 activation in inflammatory disorders, such as myocardial reperfusion injury, stroke, shock, and abdominal aortic aneurysm [9–11].

Remifentanil, a potent *μ*-opioid receptor agonist, is a synthetic ultra-short-acting opioid. It can be metabolized by nonspecific plasma and tissue esterases and is consequently unaffected by renal or liver function [12]. It has been demonstrated that remifentanil reduces IL-6 production and iNOS in septic mice [13]. Treatment with remifentanil influences neutrophil migration through endothelial cell monolayers, as well as adhesion molecule expression [14]. Moreover, remifentanil is proved to exert protective effect in a variety of diseases, such as acute lung injury, ischemia/reperfusion injury, and excitotoxic brain damage [5, 15, 16]. However, the potential role and mechanism of remifentanil in sepsis has not been yet fully investigated.

In the present study, we aimed to investigate the role and underlying mechanism of remifentanil in LPS-induced inflammation response in human aortic endothelial cells (HAECs).

## 2. Materials and Methods

All experiments in this study were approved by ethics committee of Shandong University.

### 2.1. Cell Culture

HAECs (ATCC, USA) were cultured on 24-well plates at a density of 10^5^/well in endothelial cell medium (ECM, ScienCell, CA, USA) supplemented with 5% fetal bovine serum (FBS), 100 U/mL penicillin, and 100 *μ*g/ml streptomycin at 37°C. HAECs were randomly divided into three groups: the control group, the LPS+PBS group, and the LPS+remifentanil group. In the LPS+PBS or LPS+remifentanil group, cells were pretreated with PBS or remifentanil (2.5 *μ*M, Renfu, China) [17] for 30 min, then stimulated by LPS (10 *μ*g/ml, Sigma-Aldrich, MO, USA) for another 24 h [18]. LPS was dissolved in sterile water and added in the cell culture medium. Cells were starved for 24 h before stimulation.

### 2.2. siRNA Transfection

To inhibit PARP-1 expression, HAECs were transfected with PARP-1 siRNA (sc-29437, Santa Cruz Biotechnology, CA, USA) or negative control in Opti-MEM Medium (Invitrogen, CA, USA) by use of Lipofectamine 3000 (Invitrogen). Experiments were performed 24 h after transfection.

### 2.3. Real-Time Polymerase Chain Reaction (RT-PCR)

RNA was extracted from HAECs by use of TRIzol (Invitrogen, CA, USA). Then, cDNA was analyzed by RT-PCR with iQ™ SYBR Green Supermix (Bio-Rad Laboratories, CA, USA). The primers are shown in Table 1. Amplification, detection, and data analysis involved the use of the iCycler real-time PCR system (Bio-Rad Laboratories). The real-time program included an initial denaturation period of 1.5 min at 95°C, 40 cycles at 95°C for 15 s, and 60°C for 30 s. Individual samples were run in triplicate, and each experiment was repeated for three times. The 2^−ΔΔCt^ method was used to analyze the relative changes in gene expression.

### 2.4. Western Blotting Analysis

Equal amounts of protein extracted from HAECs were separated on 10% SDS-PAGE and electro-transferred onto nitrocellulose membrane (Amersham Biosciences, NJ, USA). After being blocked with 5% nonfat milk for 2 h at room temperature, blots were washed in TBS-T 3 times for 10 min and incubated with primary antibodies at 4°C overnight. The primary antibodies were as follows: anti-*β*-actin (1 : 1000, Cell Signaling Technology, MA, USA), anti-PARP-1 (1 : 500, Sigma-Aldrich), anti-PAR (1 : 1000, Abcam, MA, USA), anti-iNOS (1 : 200, Abcam, MA, USA), anti-ICAM-1 (1 : 500; Santa Cruz Biotechnology, CA, USA), and anti-NF-*κ*B p65 (1 : 1000, Cell Signaling Technology). After being washed in TBS-T, membranes were incubated with secondary antibody for 2 h at room temperature. Signals were detected by enhanced chemiluminescence (Millipore, MA, USA) and analyzed by use of Image-Pro Plus 6.0.

### 2.5. O_2_^−^ Production

The dihydroethidium (DHE; Beyotime, Beijing) was used to measure O_2_^−^ production in HAECs as described [11]. Briefly, cells were incubated in 5 *μ*M DHE for 30 min at 37°C in a light-protected environment, then washed with ECM without FBS for 3 times. Fluorescence was acquired by use of fluorescence microscope at 535 nm.

### 2.6. Comet Assay

DNA damage was assessed by use of a comet assay kit (Trevigen, MD, USA) as previously described [19]. Comet tail length was measured by fluorescence microscopy and then analyzed using CaspLab Comet Assay Software v1.5 (Tritek Corporation, Summerduck, VA). Ten cells were analyzed in each group.

### 2.7. Immunofluorescence

HAECs were pretreated with PBS or remifentanil for 30 min, and then stimulated by LPS for another 2 h. Then, cells were fixed in 4% paraformaldehyde (Beyotime, Beijing) and permeabilized in PBS containing 0.1% Triton X-100. After being blocked with BSA for 30 min, samples were incubated with primary antibody rabbit anti-NF-*κ*B p65 (1 : 100, Cell Signaling Technology) overnight at 4°C. Alexa 488-conjugated goat anti-rabbit IgG (1 : 500, Jackson ImmunoResearch Inc., PA, USA) was used as a secondary antibody. A drop of ProLong Gold Antifade reagent with 4′,6-diamidino-2-phenylindole (DAPI; Vector Laboratories, CA, USA) was used to seal the coverslip. Images were acquired by laser scanning confocal microscopy (LSM 710, Zeiss, Germany). Data were analyzed by use of Image-Pro Plus 6.0.

### 2.8. Statistical Analysis

Data are expressed as mean ± SD. SPSS for Windows v16.0 (SPSS Inc., Chicago, IL, USA) was used for statistical analysis. Intergroup comparisons involved 2-tailed Student *t* test or one-way ANOVA. A *p* < 0.05 was considered to indicate statistical significance.

## 3. Results

### 3.1. Remifentanil Reduced Superoxide Anion Production and DNA Damage

**Figure 1:**
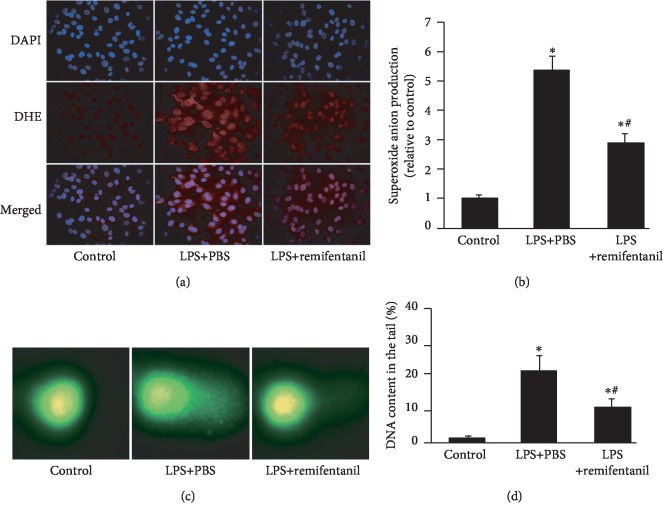
Remifentanil reduced O_2_^−^ production and DNA damage in HAECs. After pretreatment with PBS or remifentanil for 30 min, HAECs were stimulated by LPS for 24 h, superoxide anion production was measured by dihydroethidium (DHE) and DNA damage was determined by comet assay. (a, b) O_2_^−^ production (red) was assessed by DHE. Nuclei were labelled with 4′,6-diamidino-2-phenylindole (DAPI) (blue). *n* = 3. (c, d) The content of DNA in the tail was assessed by comet assay. *n* = 10. Values are means ± SD. ^∗^*p* < 0.05 versus control. ^#^*p* < 0.05 versus LPS+PBS. HAECs: human aortic endothelial cells; LPS: lipopolysaccharide.

First, we investigated the effect of remifentanil on oxidative stress. Superoxide anion production and DNA damage were analyzed by DHE staining and comet assay. The DHE staining showed that compared with the control group, LPS increased superoxide anion production, and pretreatment with remifentanil significantly reduced it (Figures 1(a) and 1(b)). As shown by comet assay in Figures 1(c) and 1(d), there is seldom DNA in the tail in the control group, while remifentanil reduced LPS-increased DNA content in the tail. These results suggested that remifentanil reduced LPS-induced oxidative stress and DNA damage.

### 3.2. Remifentanil Reduced iNOS and ICAM-1 Expressions

**Figure 2:**
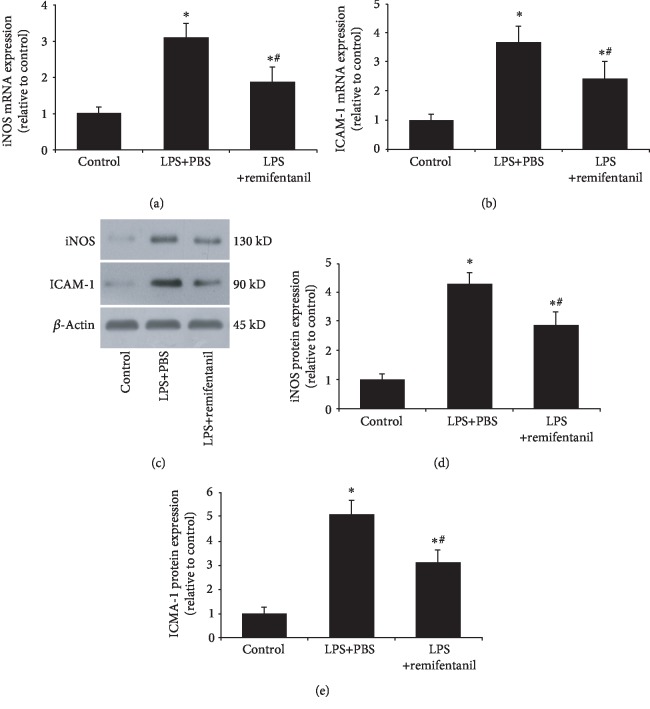
Remifentanil reduced iNOS and ICAM-1 expressions in HAECs. The mRNA and protein expressions of iNOS and ICAM-1 were assessed by RT-PCR and western blotting analysis. (a, b) RT-PCR results of iNOS and ICAM-1 mRNA expressions. (c–e) Western blot analysis of iNOS and ICAM-1 protein expressions. ^∗^*p* < 0.05 versus control. ^#^*p* < 0.05 versus LPS+PBS. iNOS: inducible nitric oxide synthase; ICAM-1: intercellular adhesion molecule 1; LPS: lipopolysaccharide. *n* = 3.

Considering the critical role of the inflammatory response in sepsis, we measured the effect of remifentanil on iNOS and ICAM-1 expressions in HAECs. RT PCR results showed that LPS stimulation markedly increased iNOS and ICAM-1 mRNA expressions as compared with the control cells. Pretreatment with remifentanil significantly reduced iNOS and ICAM-1 mRNA expressions (Figures 2(a) and 2(b)). The western blotting analysis also showed a similar result (Figures 2(c)to 2(e)). These results suggested that remifentanil could reduce LPS-induced inflammatory response.

### 3.3. Remifentanil Reduced PARP-1 Expression and Activity

**Figure 3:**
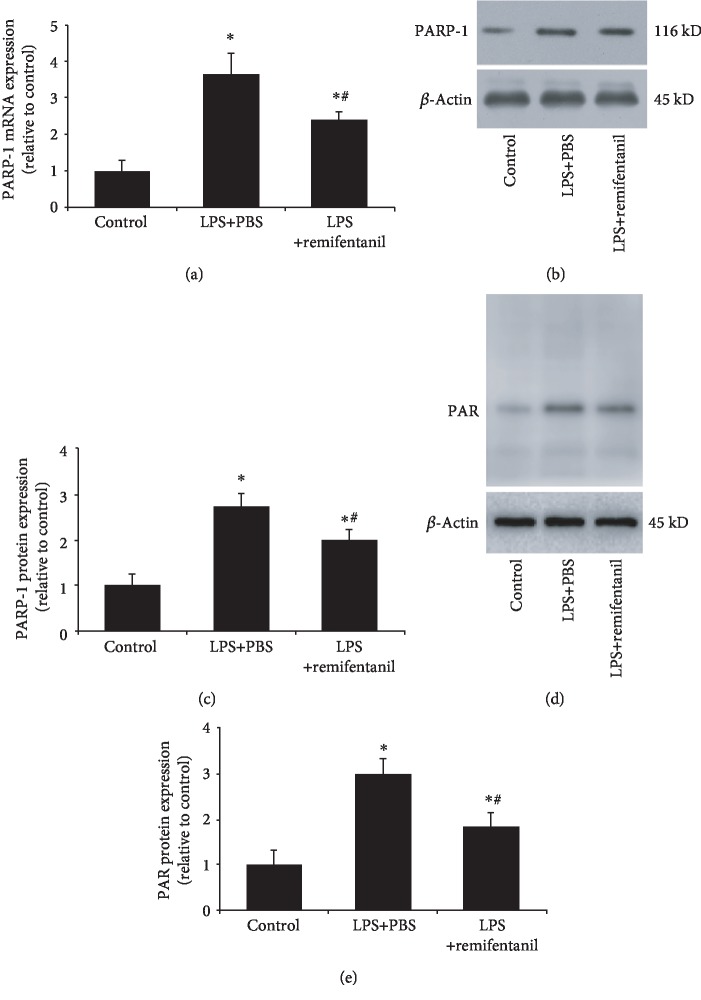
Remifentanil reduced PARP-1 expression and activity. PARP-1 expression and activity (PAR expression) were determined after stimulation. (a) RT-PCR result of PARP-1 mRNA expression. (b, c) Western blotting analysis of PARP-1 protein expression. (d, e) Western blotting analysis and quantification of PAR. Values are means ± SD. ^∗^*p* < 0.05 versus control; ^#^*p* < 0.05 versus LPS+PBS. PARP-1: poly(ADP-ribose) polymerase 1; PAR: poly(ADP-ribose); LPS: lipopolysaccharide. *n* = 3.

Then, we investigated the probable mechanism of remifentanil in anti-inflammatory effect. As a nuclear protein, PARP-1 is closely associated with the expression of various inflammatory cytokines. Thus, we firstly investigated the effect of remifentanil on PARP-1. As shown in Figure 3, LPS stimulation notably increased PARP-1 mRNA and protein expressions as well as activity (PAR expression) as compared to the control group, while remifentanil could reduce PARP-1 and PAR expressions.

### 3.4. Remifentanil Inhibited LPS-Induced NF-*κ*B p65 Nuclear Translocation and Expression

**Figure 4:**
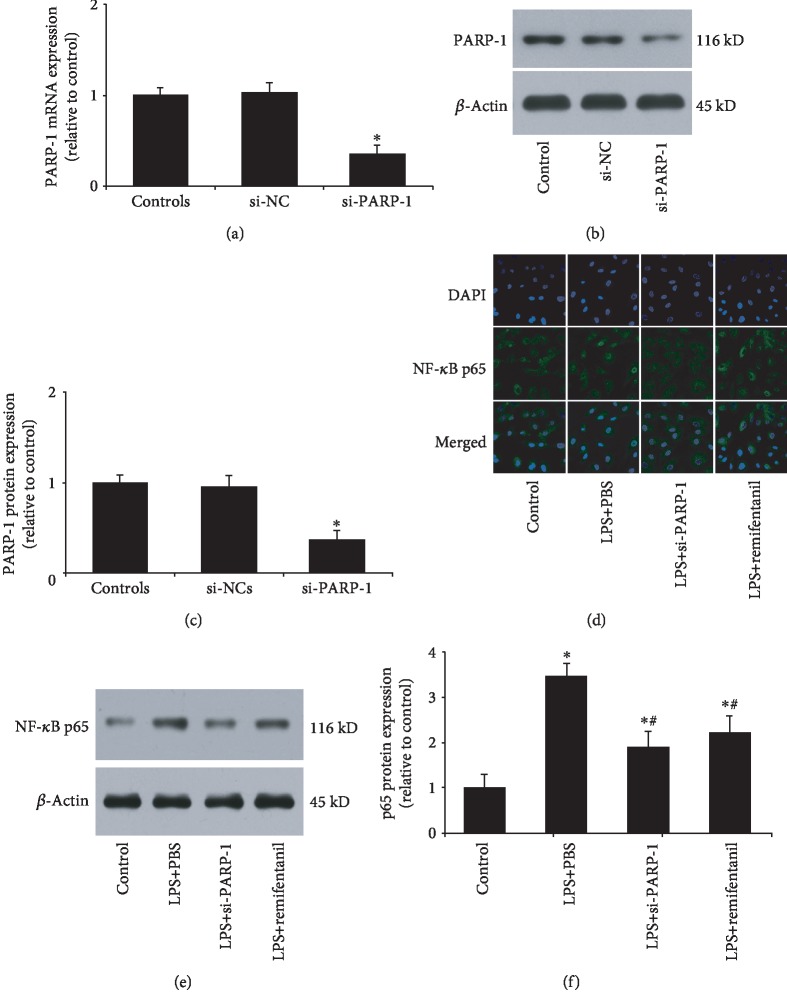
Remifentanil inhibited LPS-induced NF-*κ*B p65 nuclear translocation and expression. (a) RT-PCR of PARP-1 mRNA expression after PARP-1 inhibition by siRNA. (b, c) Western blotting analysis of PARP-1 protein expression after PARP-1 inhibition by siRNA. (d) Immunofluorescence analysis of NF-*κ*B p65 (green) and DAPI (blue). Nuclei were labelled with 4′,6-diamidino-2-phenylindole (DAPI) (blue); p65 was stained with rabbit anti-p65 primary antibody and Alexa 488-conjugated goat anti-rabbit second antibody (green). (e, f) Western blotting analysis of NF-*κ*B p65 protein expression. ^∗^*p* < 0.05 versus control, ^#^*p* < 0.05 versus LPS+PBS. si-PARP-1: siRNA of PARP-1; si-NC: negative control of PARP-1 siRNA; PARP-1: poly(ADP-ribose) polymerase 1; LPS: lipopolysaccharide. *n* = 3.

PARP-1 siRNA was used in the following experiment. First, PARP-1 expression was assessed by RT-PCT and western blotting analysis to validate the efficiency. As shown in Figures 4(a)–4(c), PARP-1 siRNA could significantly reduce the mRNA and protein expressions of PARP-1. Given the important role of NF-*κ*B in iNOS and ICAM-1 expression, we wondered whether the anti-inflammatory effect of remifentanil was associated with defective NF-*κ*B activation.  Figure 4(d) shows that NF-*κ*B p65 was mostly cytoplasmic before LPS stimulation, but its localization quickly changed to the nucleus after LPS stimulation. If HAECs were pretreated with remifentanil, p65 primarily remained cytoplasmic after LPS stimulation. Moreover, PARP-1 inhibition showed a similar effect to remifentanil. Then, we measured p65 protein expression. LPS could significantly increase p65 expression compared with the control, whereas PARP-1 siRNA or remifentanil reduced it (Figures 4(e) and 4(f)). These results suggested that remifentanil exerted anti-inflammatory effect through PARP-1/NF-*κ*B pathway.

## 4. Discussion

Our study shows that remifentanil attenuates LPS-induced oxidative stress and inflammatory response primarily by inhibition the PARP-1/NF-*κ*B signaling pathway, which may have immediate clinical implications. Remifentanil may be a superior analgesia in sepsis or septic shock.

Sepsis is known to induce the formation of reactive oxygen species and lipid peroxidation products in various target organs [20, 21]. The free oxygen radical superoxide anion (O_2_^−^) is formed from multiple sources, including dysfunctional mitochondria, xanthine oxidase, NADPH oxidase, and catecholamine auto-oxidation [22]. Remifentanil is an ultra-short-acting phenylpiperidine opioid analgesic that is rapidly metabolized by nonspecific blood and tissue esterases. In clinical practice, remifentanil is commonly used since it can be given in high doses and easily titratable, enabling fast recovery postoperatively. Remifentanil preconditioning has been shown to attenuate myocardial ischemia reperfusion injury [23, 24]. It is also demonstrated that remifentanil preconditioning reduces postischemic myocardial infarction and improves left ventricular function [17]. In our experiment, we found that LPS simulation increased superoxide anion production and induced DNA damage, while pretreatment with remifentanil could reduce the effects.

Inflammation response is an initial feature of sepsis. Attention was focused on the proinflammatory aspects of the immune response in sepsis, mostly because the typical early proinflammatory cytokines TNF and IL-1 were the first to be shown to induce organ failure in animals [1]. ICAM-1 and iNOS are also present in elevated quantities in patients with an inflammatory state, such as sepsis [25]. Several experimental data showed that the inhibition of iNOS could be an important therapeutic target to treat septic shock patients and that the adhesion molecules (such as ICAM-1) have been related with the gravity of the sepsis syndrome [26, 27]. We also found that LPS stimulation significantly increased the mRNA and protein expressions of iNOS and ICAM-1. However, remifentanil could reduce the LPS-induced expression of iNOS and ICAM-1, which suggested an anti-inflammatory role of remifentanil.

PARP-1 is a DNA repair-associated nuclear enzyme that is activated in response to DNA damage [28]. PARP-1 consumes nicotinamide adenine dinucleotide (NAD) to catalyze the addition of PAR (ADP-ribose polymers) to their target proteins, which can modify their functions, interactions, or subcellular localization [29]. Excessive activation of PARP-1 leads to intracellular depletion of NAD and ATP, thus resulting in cellular energy crisis, cytotoxicity, and even cell death [30]. Our study found that LPS could increase PARP-1 expression and activation by induction of DNA damage, while pretreatment with remifentanil reduced DNA damage, with a downregulation of PARP-1 and PAR.

The transcription factor NF-*κ*B plays a critical role in the regulation of key inflammatory genes and as a regulator of cell survival and proliferation [31]. The active binding form of NF-*κ*B is composed of various combinations of members of the NF-*κ*B/Rel family. The NF-*κ*B complex remains inactive in the cytoplasm through interaction with the inhibitory protein inhibitor *κ*B (I*κ*B). Activation of NF-*κ*B requires phosphorylation of I*κ*B*α* by I*κ*B*α* kinase and subsequent degradation [32]. In macrophages derived from PARP-1 gene knockout mice, NF-*κ*B p65 remained primarily cytoplasmic during the course of treatment with LPS [33]. In our experiment, we also found that LPS stimulation induced NF-*κ*B p65 translocation from the cytoplasm to the nucleus. However, NF-*κ*B p65 remained primarily cytoplasmic, if HAECs were pretreated with remifentanil before LPS. Meanwhile, PARP-1 inhibition by siRNA also inhibited NF-*κ*B p65 nuclear translocation. Together with our previous finding that remifentanil could reduce LPS-increased PARP-1 expression and activity, our experiment suggested that remifentanil inhibited inflammatory response through PARP-1/NF-*κ*B pathway.

There are some limitations in the experiment. We merely performed the *in vitro* experiment, and the *in vivo* as well as clinical experiment will be performed in our following experiments.

Taken together, as far as we know, this is the first time that we have demonstrated that remifentanil reduced LPS-induced oxidative stress and DNA damage, with a decreased iNOS and ICAM-1 expressions through PARP-1/NF-*κ*B signaling pathway. Remifentanil is a superior option in the use of analgesia in sepsis.

## 5. Conclusion

Our study found that remifentanil reduced LPS-induced iNOS and ICAM-1 expressions through PARP-1/NF-*κ*B signaling pathway in HAECs. Remifentanil might be a better choice of analgesia in septic patients. However, additional evidence should be provided in animal models and clinical trials.

## Figures and Tables

**Table 1:** The sequences of primers for real-time PCR.

Genes	Forward	Reverse
iNOS	5′-CGTGGAGACGGGAAAGAAGT -3′	5′-GACCCCAGGCAAGATTTGGA -3′
ICAM-1	5′-ATGGCAACGACTCCTTCTCG-3′	5′-GCCGGAAAGCTGTAGATGGT-3′
PARP-1	5′-TTGAAAAAGCCCTAAAGGCTCA-3′	5′-CTACTCGGTCCAAGATCGCC-3′
*β*-Actin	5′-CATGTACGTTGCTATCCAGGC-3′	5′-CTCCTTAATGTCACGCACGAT-3′

iNOS: inducible nitric oxide synthase; ICAM-1: intercellular adhesion molecule 1; PARP-1: poly(ADP-ribose) polymerase 1.

## Data Availability

The data used to support the findings of this study are available from the corresponding author upon request.

## References

[1] Lelubre C., Vincent J. L. (2018). Mechanisms and treatment of organ failure in sepsis. *Nature Reviews. Nephrology*.

[2] Vincent J. L., Marshall J. C., Namendys-Silva S. A. (2014). Assessment of the worldwide burden of critical illness: the intensive care over nations (ICON) audit. *The Lancet Respiratory Medicine*.

[3] Iwashyna T. J., Ely E. W., Smith D. M., Langa K. M. (2010). Long-term cognitive impairment and functional disability among survivors of severe sepsis. *JAMA*.

[4] van der Poll T., van de Veerdonk F. L., Scicluna B. P., Netea M. G. (2017). The immunopathology of sepsis and potential therapeutic targets. *Nature Reviews. Immunology*.

[5] Zhang Y., Du Z., Zhou Q., Wang Y., Li J. (2014). Remifentanil attenuates lipopolysaccharide-induced acute lung injury by downregulating the NF-*κ*B signaling pathway. *Inflammation*.

[6] Bai P. (2015). Biology of poly(ADP-ribose) polymerases: the factotums of cell maintenance. *Molecular Cell*.

[7] Kraus W. L., Lis J. T. (2003). PARP goes transcription. *Cell*.

[8] Jagtap P., Szabo C. (2005). Poly(ADP-ribose) polymerase and the therapeutic effects of its inhibitors. *Nature Reviews. Drug Discovery*.

[9] Zingarelli B., Salzman A. L., Szabo C. (1998). Genetic disruption of poly (ADP-ribose) synthetase inhibits the expression of P-selectin and intercellular adhesion molecule-1 in myocardial ischemia/reperfusion injury. *Circulation Research*.

[10] Eliasson M. J., Sampei K., Mandir A. S. (1997). Poly(ADP-ribose) polymerase gene disruption renders mice resistant to cerebral ischemia. *Nature Medicine*.

[11] Liang E. S., Bai W. W., Wang H. (2018). PARP-1 (poly[ADP-ribose] polymerase 1) inhibition protects from Ang II (angiotensin II)-induced abdominal aortic aneurysm in mice. *Hypertension*.

[12] Penido M. G., Garra R., Sammartino M., Pereira e Silva Y. (2010). Remifentanil in neonatal intensive care and anaesthesia practice. *Acta Paediatrica*.

[13] Zongze Z., Jia Z., Chang C., Kai C., Yanlin W. (2010). Protective effects of remifentanil on septic mice. *Molecular Biology Reports*.

[14] Hofbauer R., Frass M., Gmeiner B. (2000). Effects of remifentanil on neutrophil adhesion, transmigration, and intercellular adhesion molecule expression. *Acta Anaesthesiologica Scandinavica*.

[15] Zhao G., Shen X., Nan H. (2013). Remifentanil protects liver against ischemia/reperfusion injury through activation of anti-apoptotic pathways. *The Journal of Surgical Research*.

[16] Chollat C., Lecointre M., Leuillier M. (2019). Beneficial effects of remifentanil against excitotoxic brain damage in newborn mice. *Frontiers in Neurology*.

[17] Qiao S., Mao X., Wang Y. (2016). Remifentanil preconditioning reduces postischemic myocardial infarction and improves left ventricular performance via activation of the Janus activated kinase-2/signal transducers and activators of transcription-3 signal pathway and subsequent inhibition of glycogen synthase Kinase-3*β* in rats. *Critical Care Medicine*.

[18] Baumgarten G., Knuefermann P., Schuhmacher G. (2006). Toll-like receptor 4, nitric oxide, and myocardial depression in endotoxemia. *Shock*.

[19] Qin W. D., Wei S. J., Wang X. P. (1833). Poly(ADP-ribose) polymerase 1 inhibition protects against low shear stress induced inflammation. *Biochimica et Biophysica Acta (BBA) - Molecular Cell Research*.

[20] Garcia Soriano F., Liaudet L., Marton A. (2001). Inosine improves gut permeability and vascular reactivity in endotoxic shock. *Critical Care Medicine*.

[21] Liaudet L., Soriano F. G., Szabo C. (2000). Biology of nitric oxide signaling. *Critical Care Medicine*.

[22] Soriano F. G., Nogueira A. C., Caldini E. G. (2006). Potential role of poly(adenosine 5'-diphosphate-ribose) polymerase activation in the pathogenesis of myocardial contractile dysfunction associated with human septic shock. *Critical Care Medicine*.

[23] Kim H. S., Cho J. E., Hong S. W., Kim S. O., Shim J. K., Kwak Y. L. (2010). Remifentanil protects myocardium through activation of anti-apoptotic pathways of survival in ischemia-reperfused rat heart. *Physiological Research*.

[24] Chun K. J., Park Y. H., Kim J. S. (2011). Comparison of 5 different remifentanil strategies against myocardial ischemia-reperfusion injury. *Journal of Cardiothoracic and Vascular Anesthesia*.

[25] Lv X., Wang H. (2016). Pathophysiology of sepsis-induced myocardial dysfunction. *Military Medical Research*.

[26] Cobb J. P. (2001). Nitric oxide synthase inhibition as therapy for sepsis: a decade of promise. *Surgical Infections*.

[27] Amalakuhan B., Habib S. A., Mangat M. (2016). Endothelial adhesion molecules and multiple organ failure in patients with severe sepsis. *Cytokine*.

[28] Pacher P., Szabo C. (2008). Role of the peroxynitrite-poly(ADP-ribose) polymerase pathway in human disease. *The American Journal of Pathology*.

[29] Schreiber V., Dantzer F., Ame J. C., de Murcia G. (2006). Poly(ADP-ribose): novel functions for an old molecule. *Nature Reviews. Molecular Cell Biology*.

[30] Szabo C., Zingarelli B., O'Connor M., Salzman A. L. (1996). DNA strand breakage, activation of poly (ADP-ribose) synthetase, and cellular energy depletion are involved in the cytotoxicity of macrophages and smooth muscle cells exposed to peroxynitrite. *Proceedings of the National Academy of Sciences of the United States of America*.

[31] de Winther M. P., Kanters E., Kraal G., Hofker M. H. (2005). Nuclear factor *κ*B signaling in atherogenesis. *Arteriosclerosis, Thrombosis, and Vascular Biology*.

[32] Ogata N., Yamamoto H., Kugiyama K., Yasue H., Miyamoto E. (2000). Involvement of protein kinase C in superoxide anion-induced activation of nuclear factor-kappa B in human endothelial cells. *Cardiovascular Research*.

[33] Oumouna-Benachour K., Hans C. P., Suzuki Y. (2007). Poly(ADP-ribose) polymerase inhibition reduces atherosclerotic plaque size and promotes factors of plaque stability in apolipoprotein E-deficient mice: effects on macrophage recruitment, nuclear factor-kappaB nuclear translocation, and foam cell death. *Circulation*.

